# Ginkgolide B Mediated Alleviation of Inflammatory Cascades and Altered Lipid Metabolism in HUVECs via Targeting PCSK-9 Expression and Functionality

**DOI:** 10.1155/2019/7284767

**Published:** 2019-06-10

**Authors:** Gang Wang, Zhenbin Liu, Menghu Li, Yu Li, Sahir Sultan Alvi, Irfan Ahmad Ansari, M. Salman Khan

**Affiliations:** ^1^Department of Ulcer and Vascular Surgery, First Teaching Hospital of Tianjin University of Traditional Chinese Medicine, No. 314, Anshanxi Road, Nankai District, Tianjin 300193, China; ^2^Department of Pharmaceutical, First Teaching Hospital of Tianjin University of Traditional Chinese Medicine, Tianjin 300193, China; ^3^IIRC-5, Clinical Biochemistry & Natural Product Research Lab, Department of Biosciences, Integral University, Lucknow 226026, UP, India

## Abstract

The potential of oxidized-LDL (Ox-LDL) to elicit inflammatory responses in macrophages leading to the atherosclerosis (AS) progression is well known. Since proprotein convertase subtilisin/Kexin-9 (PCSK-9), the posttranslational regulator of LDL-receptor, is associated with elevated LDL in the circulation, the present report was aimed to uncover the ameliorative effects of Ginkgolide B, a terpenic lactone from* Ginkgo biloba*, against Ox-LDL-induced alterations in cholesterol metabolism in HUVECs. Consequently, our results demonstrated that incubation with Ox-LDL significantly upregulated the PCSK-9 expression in HUVECs, which was significantly downregulated, both at mRNA and protein level, after Ginkgolide B treatment via subsequent suppression of sterol element binding protein (SREBP-2) expression. Moreover, Ginkgolide B-mediated inhibition of PCSK-9 activity was also validated by* in silico* methods which revealed that it interferes the PSCK-9 interaction with LDL-receptor (LDL-R). Interestingly, Ox-LDL-induced LDL-R expression was further enhanced by Ginkgolide B treatment in HUVECs. Moreover, Ginkgolide B treatment lead to downregulation of lectin-like Ox-LDL receptor (LOX-1) and NADPH oxidase (NOX-4) expression which was upregulated in Ox-LDL-treated HUVECs, along with the attenuation of mitochondrial ROS generation. Furthermore, Ginkgolide B significantly inhibited the augmented expression of intercellular adhesion molecule-1 (ICAM-1) and vascular adhesion molecule-1 (VCAM-1) in Ox-LDL-activated HUVECs. Ginkgolide B also significantly ameliorated the inflammatory response in Ox-LDL-activated HUVECs by suppressing the expression of IL-1*α*, IL-1*β*, IL-6, CXCL-1, CXCL-2, and monocyte chemotactic protein (MCP-1), at mRNA and protein level. Our* in vitro* and* in silico* study established that Ginkgolide B alleviated the Ox-LDL-induced inflammatory cascades and altered lipid metabolism in HUVECs by suppressing the PCSK-9 and, thus, could be established as a treasured alternative therapeutic candidate in the atherosclerosis management.

## 1. Introduction

Atherosclerosis (AS), a leading cause of mortality due to vascular complications, is now considered as chronic inflammation disease of the arterial wall [1, 2]. Although the development of AS is caused by the dysfunction of a number of pathways, oxidized low-density lipoprotein (Ox-LDL) is well reckoned to mediate endothelial dysfunction as well as to accelerate the proliferation and vibrations of smooth muscle cells (SMCs), monocytes, macrophages, and fibroblasts, in addition to the oxidative stress response and cell damage of endothelial cells (ECs) [3], eventually leading to AS plaque formation. Proprotein convertase subtilisin/kexin 9 (PCSK-9), a hepatic protease, is expressed in various cells types/tissues in human and other mammals with maximum expression in hepatocytes [4, 5]. Numerous earlier reports uncovered that PCSK-9 plays a major role in cholesterol homeostasis as it elevates the LDL-C level in the circulation via degrading the LDL-receptor (LDL-R) in the hepatocyte cell membrane [5, 6]. It has also been shown to be involved in the Ox-LDL-induced apoptosis of human umbilical vein endothelial cells (HUVECs) [7].

Since LDL-C has been proved to be the most influential risk factor for onset of AS, several studies attempted to establish an association between cholesterol homeostasis and inflammation. Based on the above physiological effects, PCSK-9 is thought to be the major regulator of the LDL-C load in the circulation and hepatic cholesterol homeostasis [8, 9]. PCSK-9 has also been detected in the AS plaque and the LDL-R abundance in macrophages is negatively influenced by SMCs-derived PCSK-9 [9, 10]. Inflammation has also been found to markedly alter the lipoprotein and triglyceride-rich lipoprotein (TGRLs) metabolism as well as stimulating PCSK-9 expression [5]. More importantly, hypercholesterolemia and related cardiovascular manifestations, i.e., AS, are now widely accepted as inflammatory diseases [11].

Lectin-like Ox-LDL receptor-1 (LOX-1), a scavenger receptor, reckoned to internalize the Ox-LDL and is stimulated in distinct inflammatory complications, including AS [5, 12, 13]. A study in cultured aortic ECs and SMCs has suggested an interesting cross-talk between PCSK-9 and LOX-1 expression, as both PCSK-9 and LOX-1 stimulated each other, predominantly under inflammatory state [5].

An interaction between ECs and inflammatory cells has the capability to trigger the production inflammatory cytokines [14], whereas the PCSK-9 has also been found to elicit an inflammatory response via secretion of inflammatory cytokines and the chemokines [9]. Thus, resulting cross-talk between these cells may play a pivotal role in the development of inflammatory states. Thus, retarding the Ox-LDL-stimulated inflammatory cascades and altered lipid metabolism in ECs could be a potential line of attack in preventing the progression of AS. Ginkgolide B, a diterpenoid derived from* Ginkgo biloba*, has been known for its extensive pharmacological effects. It has been implied as a specific platelet activating factor (PAF) receptor antagonist and prevents PAF-mediated platelet activation [15, 16]. Moreover, a recent report has described the protective role of Ginkgolide B in AS, but the detailed mechanism of action of this compound including its effect on PCSK-9-LDL-R pathway and inflammation is still not elucidated completely [17]. Therefore, the present report was undertaken to assess the effect of Ox-LDL on PCSK-9-LDL-R pathway, oxidative stress, and proinflammatory cascade in HUVECs and its alleviation by Ginkgolide B.

## 2. Materials and Methods

### 2.1. Reagents and Assay Kits

Ginkgolide B and 2',7'-dichlorodihydrofluorescein diacetate (DCFH-DA) were procured from Sigma Aldrich, USA. Ambion's RNAqueous™ Total RNA Isolation Kit, Verso cDNA synthesis kit, and DyNAmo Color Flash SYBR Green qPCR Kits were bought through Thermo Fisher Scientific, USA. Dulbecco's Modified Eagle's Medium (DMEM), FBS, and other analytical-grade chemicals were purchased from HiMedia Pvt. Ltd., Mumbai, India.

### 2.2. Preparation of Ox-LDL

Human LDL was isolated as described previously and subjected to oxidation by incubation with 5 mM CuSO4 [18]. The success of LDL oxidation was confirmed by TBARS assay [19, 20]. Thus, prepared Ox-LDL was filtered and maintained at 4°C. The protein concentration of LDL was assayed using Bradford's method [21].

### 2.3. Human Umbilical Vein Endothelial Cells (HUVECs) Culture

The HUVECs were obtained from American Type Culture Collection (ATCC), USA. Cells were cultured under standard culture conditions in DMEM containing 10% heat-inactivated FBS, 2 mM glutamine, and antibiotics (100U/ml). To study the impact of oxidatively modified-LDL on various biochemical and molecular parameters, HUVECs were incubated with Ox-LDL (25-100*μ*g/ml), whereas to evaluate the antiatherogenic potential of Ginkgolide B, the cells were pretreated (24 hr) with varying concentrations of Ginkgolide B (20-100*μ*g/ml) followed by induction with Ox-LDL (75*μ*g/ml) for 24 hr.

### 2.4. RNA Extraction and Quantitative Real-Time PCR (qRT-PCR)

The Ambion's RNAqueous™ Total RNA Isolation Kit was applied to isolate the total cellular RNA (tcRNA), after harvesting the above cultured HUVECs, followed by cDNA synthesis from tcRNA using a Verso cDNA synthesis kit. qRT-PCR was run on the ABI-7500 real-time PCR machine (Applied Biosystems) using DyNAmo Color-Flash SYBR Green qPCR Kit according to the manufacturer's instructions. Specific primers used in qRT-PCR analysis have been listed in Table 1 [17, 22, 23]. GAPDH was used as internal control to normalize relative mRNA levels for all genes. Differences in the expression of various genes were calculated using 2^−ΔΔCt^ method.

### 2.5. Cholesterol Quantitation Assay

To uncover the ameliorative ability of Ginkgolide B on cholesterol accumulation in ECs, above HUVECs were collected and their total cholesterol (TC) and free cholesterol (FC) were quantified by Cholesterol Quantitation Kit supplied by Sigma-Aldrich, USA.

### 2.6. Cell Lysate Preparation

The above harvested HUVECs cells were lysed using lysis buffer, containing protease inhibitors, EDTA, and Tris-HCl followed by sonication and centrifugation at 15,000g for 5 min. Thus, obtained lysates were used for the quantification of various proteins.

### 2.7. Enzyme Linked Immunosorbant Assay (ELISA)

ELISA was used to determine the protein level of PCSK-9, cytokines (IL-1*α*, IL-1*β*, & IL-6), and chemokines (MCP-1, CXCL-1, and CXCL-2). Various ELISA kits were supplied by Abcam, United Kingdom.

### 2.8. Measurement of Intracellular ROS Generation

The intracellular ROS level was measured by previously described DCFH-DA method [24]. Briefly, 1x10^4^ cells were pretreated with Ginkgolide B (20, 40, 80, and 100*μ*g/ml) for 24 h and then treated with Ox-LDL (75*μ*g/ml) for next 6h. After incubation, the cells were treated with DCFH-DA (10*μ*M) for half of an hour at 37°C followed by measurement of fluorescence intensity (FI) (excitation and emission wavelength of 485 & 528 nm, respectively).

### 2.9. Molecular Informatics Studies of Ginkgolide B against PCSK-9 and Its Interaction with EGF-A Complex

The PCSK-9 structure was obtained from the PDB (ID: 2p4e) (http://www.rcsb.org). The sdf files of Ginkgolide B (PubChem ID: 6324617) and Atorvastatin (PubChem ID: 60823) were retrieved from PubChem database (https://pubchem.ncbi.nlm.nih.gov/). Molecular docking analysis was executed by using AutoDock (version; 4.2) [19]. EGF-A like repeats of LDL-R in complex with PCSK-9 were also retrieved from PDB (PDB ID: 3BPS). 3D conformation of PCSK-9 from this complex was removed prior to protein-protein interaction (PPI) analysis [19]. The PPI analysis of Ginkgolide B-PCSK-9 with EGF-A-like repeats of LDL-R was performed by ZDOCK server (http://zdock.umassmed.edu/).

### 2.10. Statistical Analysis

The assays were performed in triplicate and the results of these experiments have been denoted as the mean ± standard error of means (SEM). The Student's* t*-test was used to calculate p-values between two groups, whereas, for comparing multiple groups, one-way analysis of variance (ANOVA) was performed, which was followed by Post Hoc Tukey-Kramer multiple comparisons test. All the statistical analyses were carried out using GraphPad Prism version 4.02 (GraphPad Software, San Diego, USA). p<0.05, p<0.01, and p<0.001 were considered statistically significant, highly significant, and very highly significant, respectively, whereas p>0.05 was considered as statistically nonsignificant. The level of significance for each data set (with different p values) has been mentioned in respective figure legends using distinct symbols.

## 3. Results

### 3.1. Ginkgolide B Amends the Ox-LDL-Induced Altered Expression of PCSK-9, SREBP-2, and LDL-R in HUVECs

**Figure 1:**
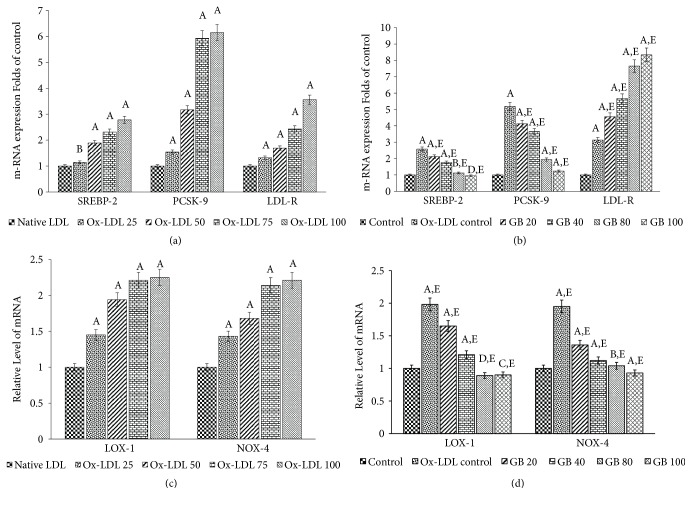
*(a) Ox-LDL alters the expression of SREBP-2, PCSK-9, and LDL-R in HUVECs.* The HUVECs were cultured in Dulbecco's Modified Eagle's Medium (DMEM) containing 10% heat-inactivated FBS, 2 mM glutamine, and antibiotics at 37°C in a humidified 5% CO_2_ atmosphere. Thus, cultured cells were either treated with native LDL or Ox-LDL at different concentrations 25, 50, 75, and 100*μ*g/ml designated as Ox-LDL 25, Ox-LDL 50, Ox-LDL 75, and Ox-LDL 100, respectively, for gene expression analysis. Significantly different from native LDL at ^A^p<0.001. Nonsignificantly different from native LDL at ^B^p>0.05.* (b) Ginkgolide B amends the Ox-LDL-induced altered expression of SREBP-2, PCSK-9, and LDL-R in HUVECs*. The HUVECs were pretreated with 20-100 *μ*g/ml of Ginkgolide B (designated as GB 20, GB 40, GB 80, and GB 100) for 24 h and then all the groups except control were exposed to Ox-LDL (75*μ*g/ml) for another 24 h followed by m-RNA extraction for qRT-PCR analysis. Significantly different from control HUVECs at ^A^p<0.001. Significantly different from control HUVECs at ^B^p<0.01. Significantly different from control HUVECs at ^C^p<0.05. Nonsignificantly different from control HUVECs at ^D^p>0.05. Significantly different from Ox-LDL at ^E^p<0.001.* (c) Ox-LDL upregulates the expression of LOX-1 and NOX-4 in HUVECs*. The HUVECs were cultured and treated with Ox-LDL as described in (a). Significantly different from native LDL at ^A^p<0.001.* (d) Ginkgolide B downregulates the Ox-LDL-induced expression of LOX-1 and NOX-4 in HUVECs*. The HUVECs were cultured and treated with Ginkgolide B and Ox-LDL as described in (b). Significantly different from control HUVECs at ^A^p<0.001. Significantly different from control HUVECs at ^B^p<0.01. Significantly different from control HUVECs at ^C^p<0.05. Nonsignificantly different from control HUVECs at ^D^p>0.05. Significantly different from Ox-LDL at ^E^p<0.001. All the values in (a), (b), (c), and (d) have been expressed as mean ± SEM of three independent experiments.

First of all, we set out to examine whether Ox-LDL affects PSCK-9 expression in HUVECs. In this context, we observed a dose-dependent overexpression of PCSK-9 in HUVECs by Ox-LDL with highest expression at 75 and 100 *μ*g/ml (5.93 and 6.15 folds of control, respectively) (Figure 1(a)). Then, we examined the ameliorative effect of Ginkgolide B on Ox-LDL-stimulated PSCK-9 overexpression in HUVECs. As shown in Figure 1(b), the incubation with Ox-LDL (75*μ*g/ml) induced an increase in PCSK-9 mRNA expression in HUVECs (5.18 folds), when matched to only DMSO treated control cells, whereas the pretreatment with 20, 40, 80, and 100 *μ*g/ml of Ginkgolide B suppressed the expression of PCSK-9 in HUVECs (up to 4.13, 3.65, 1.94, and 1.23 folds of control, respectively).

On the other hand, sterol regulatory element binding protein-2 (SREBP-2) is an important transcriptional regulator of PCSK-9 expression. Thus, we further investigated whether Ox-LDL affects SREBP-2 expression in HUVECs. Interestingly, a dose-dependent expression of SREBP-2 was induced in HUVECs by Ox-LDL and was found to be highly expressed at 75 and 100*μ*g/ml of doses (2.31 and 2.78 folds of control, respectively) (Figure 1(a)). Then, we analyzed the impact of Ginkgolide B pretreatment on the expression of SREBP-2 in the same cells incubated with oxidatively modified LDL. As shown in Figure 1(b), the incubation of HUVECs with modified-LDL (75*μ*g/ml) upregulated the SREBP-2 expression (2.58 folds), when matched to only DMSO-treated control HUVECs, while the pretreatment of cells with 20, 40, 80, and 100*μ*g/ml of Ginkgolide B downregulated the SREBP-2 mRNA expression by 2.12, 1.76, 1.12, and 0.95 folds of control, respectively. Thus, our results have also shown that Ginkgolide-mediated suppression of PCSK-9 expression was achieved via SREBP-2 downregulation in HUVECs.

Similarly, we further analyzed that whether Ox-LDL affects LDL-R expression in HUVECs and reported a dose-dependent upregulation of LDL-R expression in HUVECs by Ox-LDL with the maximum expression at 75 and 100*μ*g/ml (2.43 and 3.56 folds of control, respectively) (Figure 1(a)). Further, we determined the potential of Ginkgolide B to ameliorate the LDL-R expression also in Ox-LDL-challenged HUVECs. As shown in Figure 1(b), the incubation with Ox-LDL (75*μ*g/ml) resulted in an upregulation in LDL-R expression (3.13 folds), when equated to only DMSO-treated HUVECs, whereas the Ginkgolide B pretreatment further augmented the LDL-R expression (up to 4.56, 5.67, 7.65, and 8.34 folds of control, respectively). Thus, our findings showed an interesting phenomenon that Ginkgolide B treatment augmented the expression of LDL-R along with suppression of PCSK-9.

### 3.2. Ginkgolide B Suppressed the LOX-1 and NOX-4 Expression in HUVECs

Further, we analyzed the effect of Ox-LDL on the LOX-1 expression and reported that the LOX-1 expression was upregulated by 2.21 and 2.25 folds in 75 and 100*μ*g/ml Ox-LDL-treated cells, respectively (Figure 1(c)). Later on, we examined the protective impact of Ginkgolide B on Ox-LDL-induced LOX-1 expression. As represented in Figure 1(d), Ox-LDL upregulated the LOX-1 expression by 1.98 folds, while the Ginkgolide B pretreated cells (20, 40, 80, and 100*μ*g/ml) exhibited downregulation in the LOX-1 expression by 1.65, 1.21, 0.89, and 0.90 folds, respectively.

On the other hand, NADPH oxidase-4 (NOX-4) is a well-reckoned precursor of ROS in various cells; therefore, we determined the stimulatory role of Ox-LDL on NOX-4 expression. Similar to our assumptions, NOX-4 expression was found to be upregulated in all the Ox-LDL-treated cells with maximum upregulation of 2.14 and 2.21 folds at 75 and 100*μ*g/ml, respectively (Figure 1(c)). Then, we analyzed the modulatory impact of our test compound on Ox-LDL-triggered NOX-4 expression in the same cells. As shown in Figure 1(d), Ginkgolide B pretreatment at 20, 40, 80, and 100*μ*g/ml significantly downregulated the mRNA level of NOX-4 by 1.36, 1.12, 1.04, and 0.93 folds, respectively. Thus, our findings signify that Ginkgolide B mediated inhibition of ROS in Ox-LDL-supplemented HUVECs may be linked to the suppression of NOX-4 expression in HUVECs.

### 3.3. Ginkgolide B Inhibited the Ox-LDL-Stimulated Lipid Deposition

**Figure 2:**
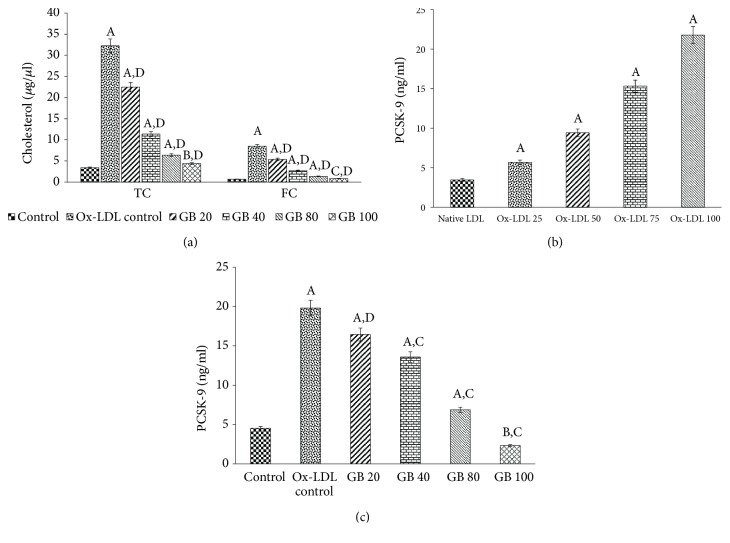
*(a) Ginkgolide B decreases Ox-LDL-induced total cholesterol and free cholesterol deposition in HUVECs*. The HUVECs were cultured in DMEM containing 10% heat-inactivated FBS, 2 mM glutamine, and antibiotics at 37°C in a humidified 5% CO_2_ atmosphere. Thus, cultured cells were pretreated with 20-100 *μ*g/ml of Ginkgolide B for 24 h and then exposed to Ox-LDL (75*μ*g/ml) for another 24 h for quantitation of TC and FC. Significantly different from control HUVECs at ^A^p<0.001. Significantly different from control HUVECs at ^B^p<0.01. Nonsignificantly different from control HUVECs at ^C^p>0.05. Significantly different from Ox-LDL at ^D^p<0.001.* (b) Ox-LDL raises the level of PCSK-9 in HUVECs*. The cultured HUVECs cells were either treated with native LDL or Ox-LDL (25, 50, 75, and 100 *μ*g/ml) for determination of PCSK-9 level by ELISA. Significantly different from native LDL at ^A^p<0.001.* (c) Ginkgolide B lowers the Ox-LDL-induced PCSK-9 level in HUVECs*. The cultured HUVECs cells were pretreated with 20-100 *μ*g/ml of Ginkgolide B for 24 h and then exposed to Ox-LDL (75*μ*g/ml) for another 24 h for quantitation of PCSK-9 via ELISA. Significantly different from control HUVECs at ^A^p<0.001. Significantly different from control HUVECs at ^B^p<0.01. Significantly different from Ox-LDL at ^D^p<0.001. Significantly different from Ox-LDL at ^E^p<0.01. All the values in (a), (b), and (c) have been expressed as mean ± SEM of three independent experiments.

In an attempt to determine the protective effect of the Ginkgolide B against Ox-LDL-induced lipid deposition in HUVECs, our data illustrated an increase in FC (from 0.68*μ*g/*μ*l to 8.49*μ*g/*μ*l), whereas the level of TC was also found to rise (from 3.41*μ*g/*μ*l to 32.25*μ*g/*μ*l) in Ox-LDL-challenged (75*μ*g/ml) HUVECs, when matched to respective control cells. Conversely, Ginkgolide B supplementation (20, 40, 80, and 100*μ*g/ml) significantly lowered the accumulation of TC and FC in HUVECs incubated with Ox-LDL (Figure 2(a)).

### 3.4. Ginkgolide B Reduces the Level of PCSK-9 in Ox-LDL-Stimulated HUVECs

We intended to uncover that whether Ox-LDL influences the protein level of PCSK-9 in HUVECs and we observed that a dose-dependent increase in the protein level of PCSK-9 induced in HUVECs by Ox-LDL with maximum PCSK-9 protein level at 75 and 100 *μ*g/ml of Ox-LDL (15.32 and 21.79ng/ml, respectively), when compared to the HUVECs treated with DMSO only (Figure 2(b)). Furthermore, the pretreatment of Ginkgolide B (20, 40, 80, and 100 *μ*g/ml) also suppressed the protein level of PCSK-9 in HUVECs with a maximum setback in 80 and 100 *μ*g/ml Ginkgolide B-treated HUVECs (Figure 2(c)).

### 3.5. Ginkgolide B Suppressed the Ox-LDL-Induced Increase of ROS Production

**Figure 3:**
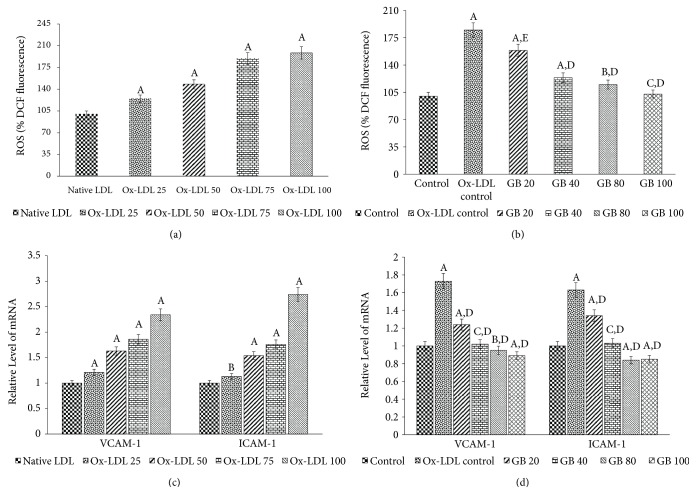
*(a) Ox-LDL treatment induces the ROS generation in HUVECs*. The cultured HUVECs cells were either treated with native LDL or Ox-LDL (25, 50, 75, and 100 *μ*g/ml) for determination of ROS generation in terms of % DCFDA fluorescence. Significantly different from native LDL at ^A^p<0.001.* (b) Ginkgolide B suppresses ROS generation in Ox-LDL-treated HUVECs*. ROS level in terms of %DCFDA fluorescence was quantified by the pretreatment of HUVECs with different concentrations of Ginkgolide B (20-100*μ*g/ml) for 24 h followed by the treatment of Ox-LDL (75*μ*g/ml) for another 6 h. Significantly different from control HUVECs at ^A^p<0.001. Significantly different from control HUVECs at ^B^p<0.01. Nonsignificantly different from control HUVECs at ^C^p>0.05. Significantly different from Ox-LDL at ^D^p<0.001. Significantly different from Ox-LDL at ^E^p<0.01.* (c) Ox-LDL treatment upregulates the expression of ICAM-1 and VCAM-1 in HUVECs*. The cultured HUVECs cells were either treated with native LDL or Ox-LDL (25, 50, 75, and 100 *μ*g/ml) for the ICAM-1 and VCAM-1 expression analysis. Significantly different from native LDL at ^A^p<0.001. Significantly different from native LDL at ^B^p<0.01.* (d) Ginkgolide B ameliorates endothelial dysfunction by suppressing expression of ICAM-1 and VCAM-1 in Ox-LDL-treated HUVECs*. Cells were pretreated with 20, 40, 80, and 100 *μ*g/ml of Ginkgolide B for 24 h and then exposed to Ox-LDL (75*μ*g/ml) for another 24 h. The cells were harvested and transcript levels of ICAM-1 and VCAM-1 were examined by qRT-PCR. Significantly different from control HUVECs at ^A^p<0.001. Significantly different from control HUVECs at ^B^p<0.01. Nonsignificantly different from control HUVECs at ^C^p>0.05. Significantly different from Ox-LDL at ^D^p<0.001. All the values in (a), (b), (c), and (d) have been expressed as mean ± SEM of three independent experiments.

In this experiment, we first determined whether Ox-LDL affects ROS production in HUVECs or not. The data, presented in Figure 3(a), showed a dose-dependent augmentation of ROS generation in Ox-LDL-treated HUVECs and was found to be raised to maximum 89.35% and 98.45%, as compared to control, at 75 and 100*μ*g/ml of doses, respectively. Further, the protective action of Ginkgolide B against Ox-LDL-induced elevation of ROS level in HUVECs was also determined. The ROS production was found to be significantly increased (up to 84.96%) in HUVECs after the exposure of Ox-LDL (75*μ*g/ml), when compared to control. As represented in Figure 3(b), the Ginkgolide B supplementation significantly diminished the Ox-LDL induced ROS production with maximum restoration in 100*μ*g/ml Ginkgolide B-treated HUVECs (2.3% of control).

### 3.6. Ginkgolide B Inhibited the Expression of Adhesion Molecules

The results, presented in Figure 3(c), showed that expression of ICAM-1 and VCAM-1 was highly expressed after incubation with Ox-LDL at doses of 75 and 100*μ*g/ml (1.76 and 2.74 folds of control for ICAM-1; 1.86 and 2.34 folds of control for VCAM-1, respectively). Moreover, an enhanced expression of ICAM-1 and VCAM-1 mRNA was observed when cells were incubated with Ox-LDL (75*μ*g/ml) by 1.63 and 1.73 folds of control, respectively. These changes in the expression of these adhesion molecules were significantly restored in Ginkgolide B treated HUVECs (Figure 3(d)). The suppression of both ICAM-1 and VCAM-1 expression by Ginkgolide B indicates that this compound could alleviate Ox-LDL-induced endothelial dysfunction.

### 3.7. Ginkgolide B Suppressed the Ox-LDL-Induced Expression of Inflammatory Cytokines in HUVECs

**Figure 4:**
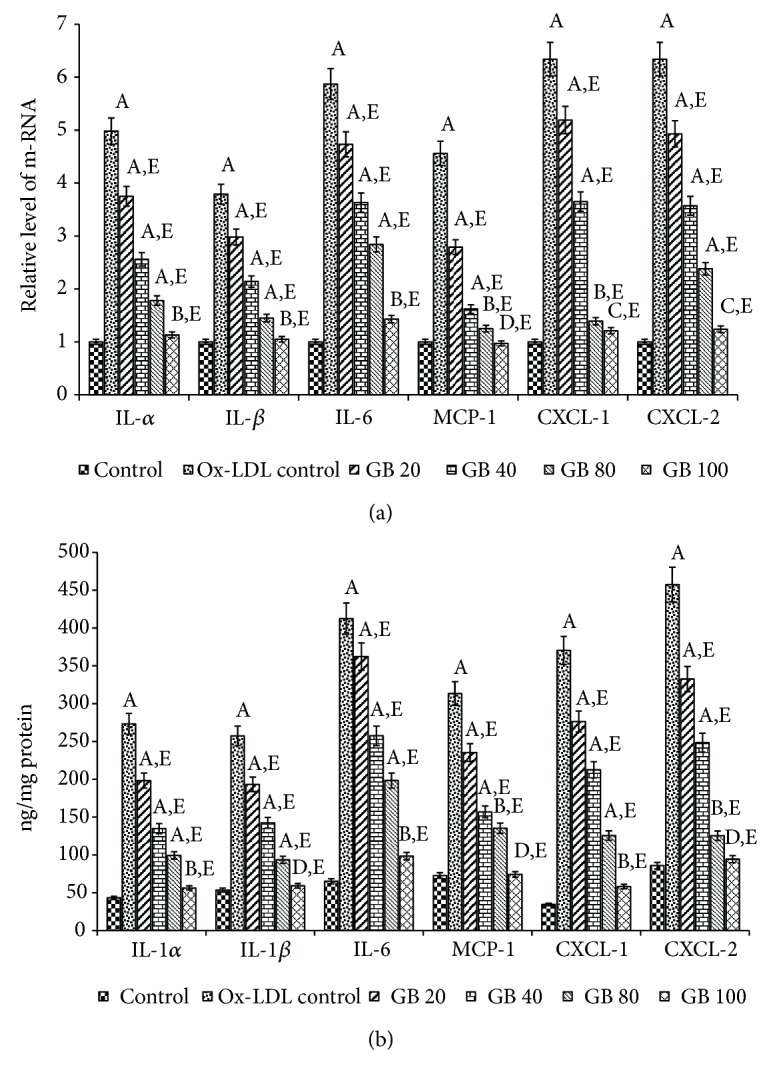
*(a) Ginkgolide B downregulates the expression of inflammatory cytokines and chemokines in Ox-LDL-treated HUVECs*. HUVECs were grown in six-well plates for 24 h and treated with different concentrations of Ginkgolide B (20-100 *μ*g/ml) for 24 h followed by the treatment of Ox-LDL (75*μ*g/ml) for another 24 h. The cells were harvested and the expression of cytokines (IL-1*α*, IL-1*β*, & IL-6) and chemokines (MCP-1, CXCL-1, & CXCL-2) was examined by qRT-PCR analysis.* (b) Ginkgolide B inhibited inflammatory protein expression in Ox-LDL-treated HUVECs*. Cells were grown in six-well plates for 24 h and treated with different concentrations of Ginkgolide B (20-100 *μ*g/ml) for 24 h followed by the treatment of Ox-LDL (75*μ*g/ml) for another 24 h. The cells were harvested and cell lysates were prepared. Protein levels of different cytokines (IL-1*α*, IL-1*β*, & IL-6) and chemokines (MCP-1, CXCL-1, & CXCL-2) were examined by ELISA kits. The levels of statistical significance for both (a) and (b) are significantly different from control HUVECs at ^A^p<0.001. Significantly different from control HUVECs at ^B^p<0.01. Significantly different from control HUVECs at ^C^p<0.05. Nonsignificantly different from control HUVECs at ^D^p>0.05. Significantly different from Ox-LDL at ^E^p<0.001. All the values in both (a) and (b) have been expressed as mean ± SEM of three independent experiments.

We also investigated the impact of Ox-LDL-mediated oxidative stress on the expression of various cytokines, i.e., Interleukins (IL)-1*α*, 1*β*, and 6 as well as chemokines, i.e., monocyte chemotactic protein (MCP-1) and chemokine (C-X-C motif) ligand (CXCL-1 and 2). The incubation with Ox-LDL (75*μ*g/ml) resulted in the overexpression of (IL)-1*α*, 1*β*, 6, MCP-1, CXCL-1, and CXCL-2 by 4.98, 3.79, 5.87, 4.56, 6.34, and 5.47 folds, respectively. Such elevations in the mRNA expression of inflammatory mediators were markedly restored in Ginkgolide B pretreated cells in comparison to untreated cells (Figure 4(a)). On the other hand, the protein level of these cytokines was found to be upregulated in HUVECs after incubation with Ox-LDL (75*μ*g/ml), assayed by ELISA. The protein level of the above-mentioned inflammatory mediators was greatly normalized in Ginkgolide B pretreated cells (Figure 4(b)).

### 3.8. Ginkgolide B Alters PCSK-9 Interaction with EGF-A

**Figure 5:**
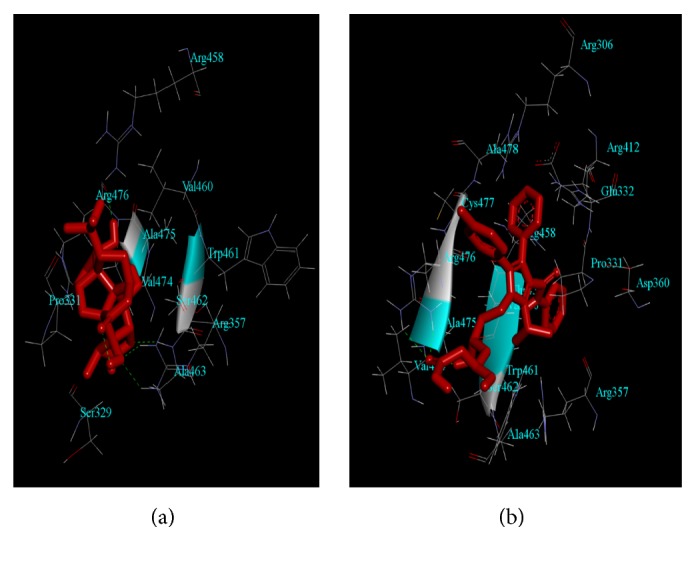
*Molecular informatics studies of Ginkgolide B and Atorvastatin with PCSK-9*.* (a)* Interaction of Ginkgolide B (represented as red ball-stick model) with PCSK-9 (PDB I.D.: 2p4e) residues showed the binding energy (ΔG) of –7.13 Kcal/mol.* (b)* Interaction of Atorvastatin (represented as red ball-stick model) with the residues of PCSK-9 showed the binding energy (ΔG) of –7.02 Kcal/mol.

**Figure 6:**
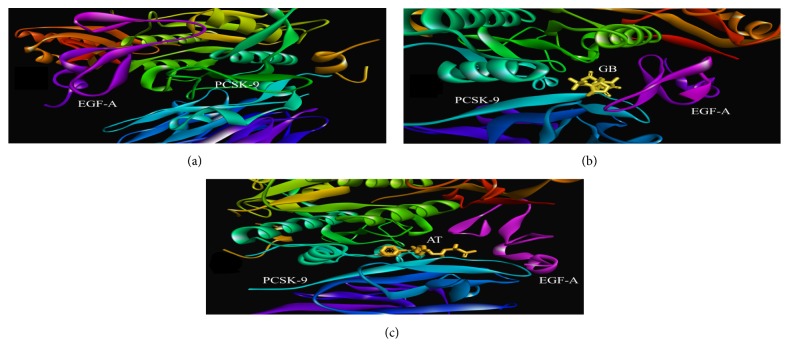
*Ginkgolide B reduces the affinity of PCSK-9 to complex with EGF-A-like repeats of LDL-R*.* (a)* PCSK-9-EGF-A-complex (PCSK-9 chains have been represented as ribbon structures in various colors, while EGF-A of LDL-R has been represented as purple ribbon-like structure).* (b)* Ginkgolide B-PCSK-9-EGF-A-complex (Ginkgolide B has been represented in yellow as ball-stick molecule).* (c)* Atorvastatin-PCSK-9-EGF-A-complex (Atorvastatin has been represented in yellow as ball-stick molecule).

Our computational approach demonstrated that the binding energies (ΔG values) for Ginkgolide B and Atorvastatin, when complexed with PCSK-9, were -7.13 and -7.02 Kcal/mol, respectively, whereas the inhibition constant was found to be 5.92 and 7.16 *μ*M, respectively. The binding of Ginkgolide B with the active site of PCSK-9 involved its interaction with the Ser329, Pro331, Arg357 Arg458, Val460, Trp461, Ser462, Ala463, Val474, Ala475, and Arg476 amino acid residues (Figure 5(a)), whereas Atorvastatin interaction with PCSK-9 involved the interaction with Arg306, Pro331, Glu332, Arg357, Asp360, Arg412, Arg458, Val460, Trp461, Ser462, ALA463, Val474, Ala475, Arg476, Cys477, Ala478, and Thr459 amino acid residues (Figure 5(b)).The most important finding from our* in silico* study was the fact that both the ligands (Ginkgolide B and Atorvastatin) were surrounded by nine common amino acid residues of PCSK-9 (Arg357, Arg458, Val460, Trp461, Ser462, ALA463, Val474, Ala475, and Arg476). Furthermore, these complexes of Ginkgolide B and Atorvastatin with PCSK-9 were used to interact with EGF-A portion of LDL-R via PPI studies, which demonstrated that Ginkgolide B destabilizes the PCSK-9-EGF-A complex (ZDOCK Score: 1031.026), whereas Atorvastatin aids in stabilization of PCSK-9-EGF-A complex (ZDOCK Score: 1071.570), when the ZDOCK Score for both these compounds was compared with PCSK-9-EGF-A-complex without any ligand (ZDOCK Score: 1055.178) (Figure 6).

## 4. Discussion

Atherosclerosis is reckoned as a chronic inflammatory ailment and is greatly assisted by vascular EC dysfunction [3, 11, 25]. PCSK-9 regulates the receptor-mediated LDL internalization into hepatocytes and subsequent atherogenic load in the vessels [26, 27]. Genetic defects in PCSK-9 lead to the establishment of familial hypercholesterolemia which ultimately progresses into AS [28, 29]. Following its synthesis and secretion, PCSK-9 combines to extracellular EGF-A domain of LDL-R and facilitates the lysosome-mediated catalysis of LDL-R, resulting in lesser no. of LDL-Rs available on hepatocytes for further LDL clearance [19, 30–32]. Together with these findings, other reports also advocate the ability of PCSK-9 to regulate the circulatory LDL-C [33–35].

It is also well reckoned that both PCSK-9 and LDL-R are coexpressed in many cell types and their gene transcription is under control of SREBP-2 [35–37]. Therefore, in the current study, we did find a decline in the PCSK-9 expression by Ginkgolide B supplementation in HUVECs. In a recent study, Ox-LDL has been found to stimulate PCSK-9 expression, indicating that perhaps inflammatory response aids in the earlier PCSK-9 activation [5]. Very few natural products, i.e., berberine and lycopene, have been reported to influence the expression of hepatocyte nuclear factor-1*α* (HNF-1*α*) and SREBP-2, thereby regulating the PCSK-9 menace [35, 38]. Thus, in accordance with the previous studies, we also concluded that Ginkgolide B exerts protective effect against atherosclerosis via limiting the PCSK-9 expression. Furthermore, we observed a decline in Ox-LDL-induced expression of SREBP-2 in Ginkgolide B-treated HUVECs which could be correlated with the decline in PCSK-9 expression in HUVECs.

Additionally, our results have also shown an interesting phenomenon that Ginkgolide B treatment augmented the expression of LDL-R along with suppression of PCSK-9 which was in contradiction to reports where statins upregulated the expression of both PCSK-9 and LDL-R, which ultimately limited the beneficial effects of statins [19, 38]. Moreover, we have also shown that Ginkgolide B treatment leads to inhibition of SREBP-2, which is responsible for the regulation of both PCSK-9 and LDL-R gene. On the other hand, the pharmacological effect of statins is achieved by upregulation of both PCSK-9 and LDL-R expression as their expression is governed by a common motif, sterol regulatory element (SRE-1), located in the LDL-R and PCSK-9 promoters, limiting the beneficial effect of statins to lower LDL-C level [35, 39–41]. Interestingly, in our study, the downregulation of SREBP-2 did not suppress/affect the LDL-R expression in HUVECs, rather it was augmented by Ginkgolide B treatment; thus, the result suggested that Ginkgolide B could regulate the LDL-R expression in HUVECs by a sterol-independent mechanism. These findings are in well agreement with previous reports [19, 38]. These findings demonstrate that Ginkgolide B exerts potent antiatherogenic effects via modes distinct to that of statins.

Moreover, the results from our* in silico* analysis did show that Ginkgolide B possesses the ability to interact with PCSK-9 in a somewhat comparable manner as atorvastatin does with nine common hydrophobic residues, i.e., Arg357, Arg458, Val460, Trp461, Ser462, ALA463, Val474, Ala475, and Arg476. Furthermore, the binding energies for both compounds (ΔG: -7.13 and -7.02 Kcal/mol, respectively) also confirmed their comparable interaction against PCSK-9 binding region. Further, our PPI studies showed very surprising results that Ginkgolide B destabilizes/prevents the PCSK-9-EGF-A complex, while atorvastatin facilitated the binding of PCSK-9 with EGF-A portion of LDL-R. This data is in well accordance with the previously published report demonstrating the ability of Atorvastatin to stimulate PCSK-9 to make a more stable PCSK-9-EGF-A-complex than it does in the absence of Atorvastatin [19].

Overexpression of LOX-1 in distinct physiological circumstances induces AS progression in hyperlipidemic mice via enhanced rate of Ox-LDL uptake and subsequent endothelial dysfunction [42]. Conversely, mice, lacking LOX-1 gene, have shown to be negatively associated with the incidence of the atherosclerosis regardless of their high-cholesterol-rich diet [12]. In order to cope with these consequences, we assessed the protective role of Ginkgolide B in Ox-LDL-triggered LOX-1 expression and reported that it significantly diminishes the LOX-1 expression in HUVECs. Furthermore, we also observed a low accumulation of TC and FC in Ox-LDL-induced cells when pretreated with Ginkgolide B which could be attributed to the diminished expression and activity of LOX-1. Our results have shown that both PCSK-9 and LOX-1 expression is enhanced in HUVECs by Ox-LDL stimulus. Ding et al. also showed a cross-talk between PCSK-9 and LOX-1 that may be of great importance in predicting atherogenic manifestations [5]. Similarly, upon PCSK-9 knockdown or knockout, LOX-1 was inhibited significantly [12]. Thus, PCSK-9 inhibition strategies could also inactivate LOX-1 which was well justified with a study that established the attenuation of Ox-LDL induced both PCSK-9 & LOX-1 expression [5], which further suggested the therapeutic importance of this compound in AS.

Furthermore, Ox-LDL is well reckoned to promote ROS generation and in the same vein, NOX-4 is also known to generate ROS, an established risk factor for AS [43, 44]. NOX-4 is highly expressed in different cells including ECs [45]. Similarly, we also reported that incubation with oxidatively modified-LDL markedly upregulated the NOX-4 expression. The results in turn illustrated that Ox-LDL promoted the expression of PCSK9, affected LDLR-mediated signaling pathway, participated in oxidative stress, and induced inflammatory cascade reaction at different levels. It is suggested that there is cross-regulation between molecules. Further, Ginkgolide B significantly restored the NOX-4 expression along with the diminished ROS level in HUVECs. Based on these findings, we refer that Ginkgolide B-mediated low level of ROS might be attributed to its ability to suppress NOX-4 mRNA, which has been in well agreement with previous report [46]. Our results showed that Ginkgolide B could weaken the effect of Ox-LDL in the whole process, and Ginkgolide B affected the function of PCSK9.

Activation of ECs induces several adhesion proteins, i.e., MCP-1, ICAM-1, and VCAM-1. On the other hand, uptake of Ox-LDL by macrophages leads to their transformation into the foam cells, which are reckoned to retrigger inflammatory cascades, leading to endothelial dysfunction and subsequent atherosclerosis plaque formation [14, 47–49]. The results of our study have clearly documented that overexpression of adhesion molecules ICAM-1 and VCAM-1 on ECs induced by Ox-LDL was attenuated by Ginkgolide B treatment as well as the expression of ILs, MCP-1, CXCL-1, and CXCL-2 was also kept in check by Ginkgolide B. It is revealed that Ginkgolide B improves the inflammatory cascade reaction activated by Ox-LDL by regulating PCSK9-LDLR signaling pathway.

PCSK-9 inhibition either by knockdown approaches or mice lacking this gene has been found to resist against the Ox-LDL as well as lipopolysaccharide (LPS) triggered systemic responses [2, 50]. On the other hand, overexpression of PCSK-9 was found to be associated with robust inflammatory response to LPS in macrophages [51]. The above findings uncovered the role of PCSK-9 in the atherosclerotic plaque formation via enhanced localized inflammation, monocyte infiltration, and monocyte transformation. Since we have shown that Ox-LDL can stimulate HUVECs to secrete PCSK-9 abundantly, thus, we can deduce an interesting inference from our results that activation and overexpression of PCSK-9 might participate in the initiation of the above observed inflammatory cascade in Ox-LDL-stimulated HUVECs. Conclusively, the results of our current study signify that Ginkgolide B-mediated inhibition of PCSK-9 expression in HUVECs could suppress these proinflammatory cascades in the atherosclerotic plaques and, thus, could possibly play therapeutic role in the alleviation of sufferings, up to some part, from AS.

## 5. Conclusion

In conclusion, our* in vitro* findings strongly suggested that Ginkgolide B could potentially suppress PCSK-9 along with SREBP-2 expression and, conversely, could augment the LDL-R expression. Ginkgolide B also inhibits LOX-1 expression and unusual lipid accumulation in ECs as well as significantly decreases NOX-4 expression and ROS generation in ECs. Moreover, Ginkgolide B also downregulated the expression of ICAM-1 and VCAM-1 in ECs and exhibited potent anti-inflammatory activity in Ox-LDL-induced HUVECs. Furthermore, our PPI studies demonstrated that Ginkgolide B destabilizes the PCSK-9-EGF-A complex, whereas Atorvastatin stabilizes the PCSK-9-EGF-A complex, when the ZDOCK scores for these compounds were compared with PCSK-9-EGF-A-complex without any ligand. Finally, owing to its protective role against the progression of atherosclerosis, further* in vivo *studies are warranted to establish its clinical significance.

## Figures and Tables

**Table 1:** Primers used in this study for gene expression analysis.

Gene name	Primer	Sequences (from 5' to 3')
SREBP-2	F	CCCTGGGAGACA TCGACGA
	R	CGTTGCACTGAAGGGTCCA

PSCK-9	F	CCTGCGCGTGCTCAACT
	R	GCTGGCTTTTCCGAAACTCT

LDL-R	F	GTGTCACAGCGGCG
	R	CGCACTCTTTGATG

NOX-4	F	TGTTGGATGACTGGAAACCA
	R	TGGGTCCACAACAGAAAACA

LOX-1	F	GCTGCTATGACTCTGGTCAT
	R	TACGATCCTGCTGAGTAAGG

ICAM-1	F	GGCCGGCCAGCTTATACAC
	R	TAGACACTTGAGCTCGGGCA

VCAM-1	F	TCAGATTGGAGACTCAGTCATGT
	R	ACTCCTCACCTTCCCGCTC

IL-1*α*	F	TTCCCTCAACCAAACTATAT
	R	ACGGGCTGGTCTTCTCCTTG

IL-1*β*	F	CTCATTGTGGCTGTGGAGAA
	R	CACACACCAGCAGGTTATCA

IL-6	F	TCTCTCCGCAAGAGACTTCCA
	R	ATACTGGTCTGTTGTGGGTGG

MCP-1	F	TTCTGTGCCTGCTGCTCATA
	R	CAGATCTCCTTGGCCACAAT

CXCL-1	F	TCATCGAAAAGATGCTGAACA
	R	TTCAGGAACAGCCACCAGT

CXCL-2	F	CATCGAAAAGATGCTGAAAAATG
	R	TTCAGGAACAGCCACCAATA

GAPDH	F	CAACAGCCTCAAGATCATCAGCA
	R	TGGCATGGTCTGTGGTCATGAGT

## Data Availability

The data used to support the findings of this study are available from the corresponding author upon request.
